# Differential expression of Cathepsin S and X in the spinal cord of a rat neuropathic pain model

**DOI:** 10.1186/1471-2202-9-80

**Published:** 2008-08-12

**Authors:** Anna Leichsenring, Ingo Bäcker, Wiebke Wendt, Michael Andriske, Beate Schmitz, Christine C Stichel, Hermann Lübbert

**Affiliations:** 1 Department of Animal Physiology, Ruhr-University of Bochum, Bochum, Germany; 2 Biofrontera Bioscience GmbH, Leverkusen, Germany

## Abstract

**Background:**

Ample evidence suggests a substantial contribution of cellular and molecular changes in the spinal cord to the induction and persistence of chronic neuropathic pain conditions. While for a long time, proteases were mainly considered as protein degrading enzymes, they are now receiving growing interest as signalling molecules in the pain pathology. In the present study we focused on two cathepsins, CATS and CATX, and studied their spatiotemporal expression and activity during the development and progression of neuropathic pain in the CNS of the rat 5^th ^lumbar spinal nerve transection model (L5T).

**Results:**

Immediately after the lesion, both cathepsins, CATS and CATX, were upregulated in the spinal cord. Moreover, we succeeded in measuring the activity of CATX, which was substantially increased after L5T. The differential expression of these proteins exhibited the same spatial distribution and temporal progression in the spinal cord, progressing up to the medulla oblongata in the late phase of chronic pain. The cellular distribution of CATS and CATX was, however, considerably different.

**Conclusion:**

The cellular distribution and the spatio-temporal development of the altered expression of CATS and CATX suggest that these proteins are important players in the spinal mechanisms involved in chronic pain induction and maintenance.

## Background

Neuropathic pain is one type of chronic pain and originates by definition from a lesion of the nervous system (for reviews see [1,2]). It is a devastating and difficult to manage disease mainly because the underlying mechanisms are still poorly understood. Indeed, several types of cells and highly complex interactions of multiple pathways have been implicated in the pathogenesis (for reviews see [1,3,4]). In this context work on animal models has emphasized the important contribution of differences in protein expression to neuropathic pain induction and maintenance (for reviews see [5-7]). Within this cocktail proteases are receiving growing interest [8-12] because of their enormous destructive potential and the irreversibility of their action (for reviews see [13-16]).

In our study we focus on a distinct group of proteases, the cathepsins (CAT), which are cysteine proteases mainly localized in lysosomes/peroxisomes but are also found in extralysosomal sites [17]. There are 11 human members (cathepsins B, C, F, H, K, L, O, S, V, W and X) and in mouse 8 additional members (cathepsins 1, 2, 3, 6, J, M, Q and R) in this group of enzymes [18,19]. They play a vital role in normal cellular protein metabolism such as the regulation of key protein kinases and phosphatases, and the induction of specific cytoskeletal rearrangements, which may account for their involvement in intracellular signaling, vesicular trafficking, and structural stabilization [20,21]. Hence, it is not suprising that CAT are implicated in the manifestation of a number of diseases, including cancer, arthritis, Morbus Parkinson, Morbus Alzheimer and age-dependent inflammation [22-27]. Recent studies suggest that the activation or breakdown of the endosomal/lysosomal proteolytic system might also be involved in pain pathophysiology. Thus, in different chronic animal pain models an upregulation of some members (S, B, H, L, D) of the CAT family along the ascending nociceptive pathway has been reported [9,10] and CATS has even been implicated in neuropathic hyperalgesia and allodynia [11,28].

With these strong implications for a role of CAT in neuropathic pain pathogenesis in mind we decided to study the spatiotemporal expression pattern of two of these proteases, the CATS and the more recently identified CATX, during the phases of pain induction and maintenance in a rat neuropathic pain model, the transection of the 5^th ^lumbar spinal nerve. CATS is a well-described cathepsin originally identified from lymph nodes and spleen [29,30] and is well known for its crucial function in the control of antigen presentation [31]. CATX, on the other hand, has only recently been localized in the central nervous system [26] and its expression pattern in pathological situations implies a role in degenerative processes [27,32].

## Results

### Cellular and spatiotemporal expression of CATS and CATX in normal and L5T spinal cord

**Figure 1:**
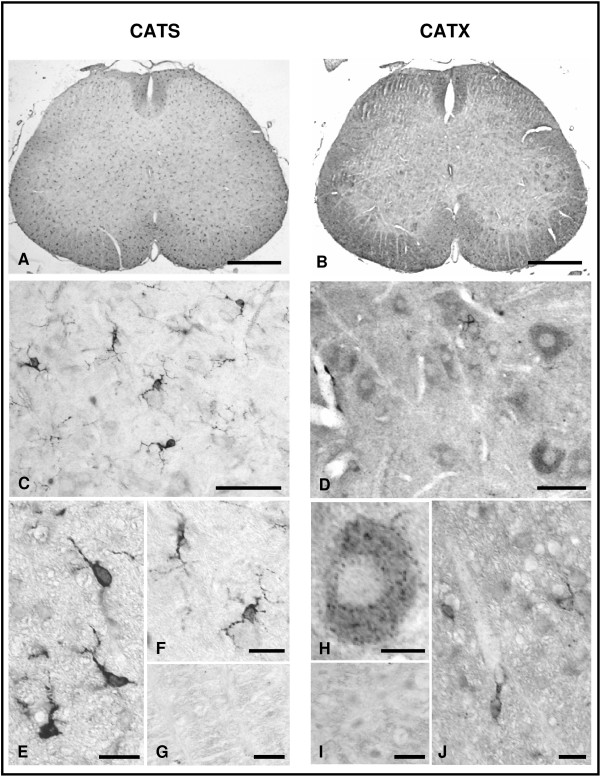
**CATS- and CATX-immunohistochemistry in normal rat spinal cord**. Representative examples of CATS- (A, C, E-G) and CATX-immunostained (B, D, H-J) sections of the L5 segment. CATS-immunopositive deposits are localized in small glial-like cells (C, E, F) that distributed homogenously throughout the section (A), while CATX is mostly found in large neurons (D, H) and only few small cells are intensely stained (D, J). G, I: Sections incubated with preabsorbed primary antibodies are free of immunostaining. Scale bars, 500 μm (A, B), 50 μm (C, D), 20 μm (E-J).

In unlesioned adult rats CATS- and CATX-immunoreactivities were found in cells of both grey and white matters (Fig. 1) throughout the entire length of the spinal cord. Most CATS-immunopositive cells were of small size and distributed uniformely (Fig. 1A, C, E, F). While CATS-immunopositive neurons were rare, CATX-immunoreactivity was found in nearly all neurons (Fig. 1B, D, H) and only in few small cells (Fig. 1D, J). The immunoreactivities were associated with spherical granules within the cytoplasm of cells, sparing the nucleus (Fig. 1E, D, H).

**Figure 2:**
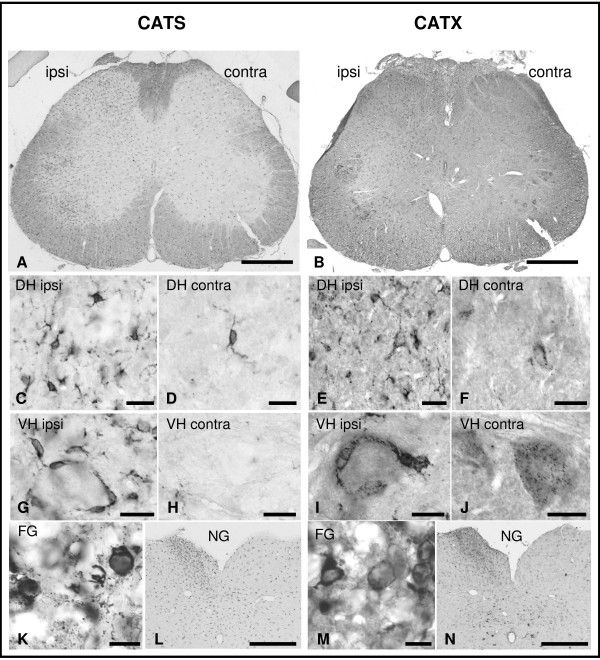
**Upregulation of CATS- and CATX-immunoreactivities in the spinal cord at 14 d after L5T**. Survey micrographs illustrate the ipsilateral increase of CATS- and CATX-immunoreactivity in whole spinal cord sections (A, B), in the dorsal horn (DH) (C-F), the layer IX of the ventral horn (VH) and the fasciculus gracilis (FG). In the FG immunopositive cells exhibit macrophage-like morphology (K, M) and in the VH small immunopositive cells engulf motoneurons (G, I). At this time point the ipsilateral nucleus gracilis exhibits more intense CATS- (L) and CATX-staining (N) than the contralateral side. Scale bars, 500 μm (A, B, L, N), 20 μm (C-J), 10 μm (K, M).

**Figure 3:**
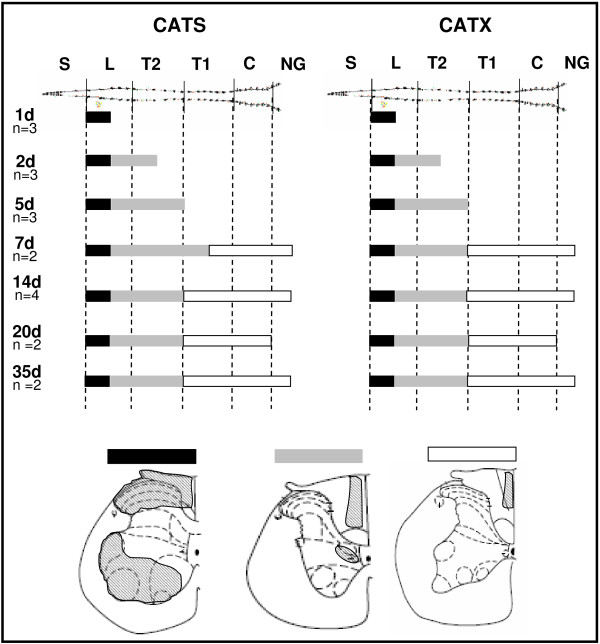
**Spatiotemporal progression of CATS-/CATX-immunoreactivities in the spinal cord after L5T**. Cranial progression of cathepsin upregulation during the first 5 weeks after transection. The different expression patterns in the transverse plane are symbolized by different fillings of the bars. Both cathepsins exhibited the same spatial and temporal distribution pattern up to 35 d after transection.

The first changes of CATS- and CATX-immunoreactivities were already notable 1 d after L5T. For both proteins we observed upregulations that were restricted to the ipsilateral fasciculus gracilis, the dorsal horn and layer IX in the ventral horn in the lumbar segment (Figs. 2 and 3). Within these regions, the number of small CATS- as well as CATX-immunopositive cells increased substantially (Fig. 2). Moreover, we found a numerical increase in CATS-immunopositive neurons, while the number of CATX-immunopositive neurons was constant. Interestingly, numerous small CATS as well as CATX-immunopositive cells engulfed large motoneurons in the ventral horn (Fig. 2G, I). Within the following days the ipsilateral increase in immunopositive cells in the fasciculus gracilis spread caudocranially to the upper SC segments and reached the gracile nucleus at 1 w after injury (Figs. 2L, N and 3). At that time-point this nucleus exhibited morphological signs of degeneration (data not shown).

### Upregulation of CATS and CATX protein levels and increase in enzyme activities

We next demonstrated that the changes in CATS- and CATX-immunoreactivities are reflected by changes in the levels of the respective proteins and, above all, that these are also reflected by a change of activity. Therefore, we first analyzed the protein levels of CATX and CATS in the spinal cord of sham versus L5T animals at 8 d after injury, a time point when the increase in immunoreactivities in the spinal cord was at its maximum (Fig. 3).

**Figure 4:**
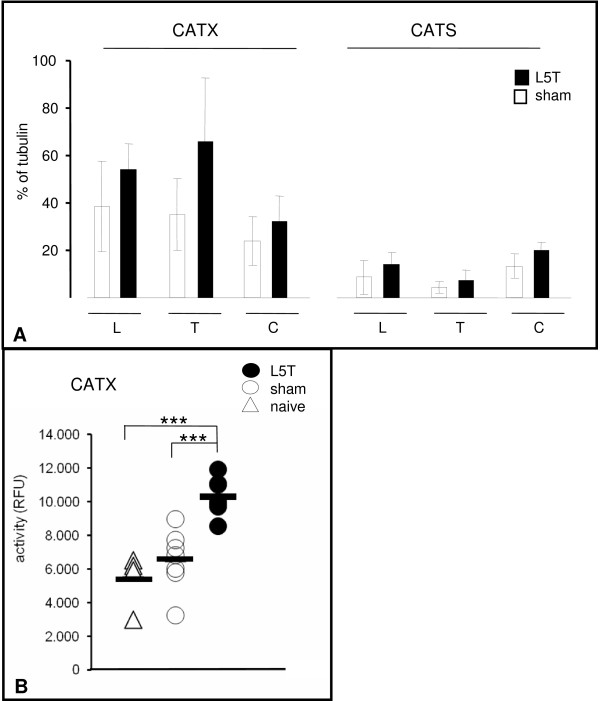
**Upregulation of cathepsin protein levels and activities after L5T**. **A: **Western blot analysis of CATX and CATS proform expression in the spinal cord of L5T (n = 5) and sham operated animals (n = 5) at 8 d after injury. Expression levels were normalized relative to the corresponding α-tubulin band. At this time point the L5 transection induced an upregulation of both proteins in all SC segments. The expression level of CATX was substantially higher than that of CATS. Data are means ± SD. **B: **CATX activities in the lumbar SC 8 d after transection in L5T, sham (n = 7, for each group) and naive animals (n = 4). Each symbol represents the value of a single animal, the bar indicates the mean for the group. CATX activity was significantly higher in L5T than in sham or naive SC. *** p ≤ 0.001. L, lumbar; C, cervical; T, thoracic.

Western blot analysis (n = 5 per group) revealed the proforms of CATS (37 kD) and CATX (34 kD), as the most prominent bands, while the prepro- and mature forms were below the detection level. The proforms of both enzymes were detected in all segments analyzed (L, lumbar; T, thoracic; C, cervical) of the adult rat spinal cord (Fig. 4A). The L5 nerve transsection produced an upregulation in all SC segments for CATS as well as CATX (Fig. 4A). The strongest increase in protein content was found in the T segment for both enzymes (CATS 63.6%; CATX 87.4%), while the increase in the L and C-segments was substantial (34–61.5%) but lower than in the T segment (Fig. 4A). Moreover, our Western blot analysis showed that in all SC segments the level of CATX is more than twice as high as the level of CATS. These results were confirmed in a second experiment with 4 animals per group (see Additional files 1 and 2).

This data is supported by measurements of CATX activity. CATS activity assays were not performed since the assay suffered from the lack of a specific substrate and a specific commercially available inhibitor for the enzyme, leaving doubts about the specificity of the assay in complex protein mixtures. At 8 d after L5T CATX activity increased strongly and highly significant (p ≤ 0.001) in the lumbar segment (Fig. 4B; 59% compared to sham, 90% compared to naive). CATB activity was not significantly changed (data not shown).

### Characterisation of CATS- and CATX-expressing cells

**Figure 5:**
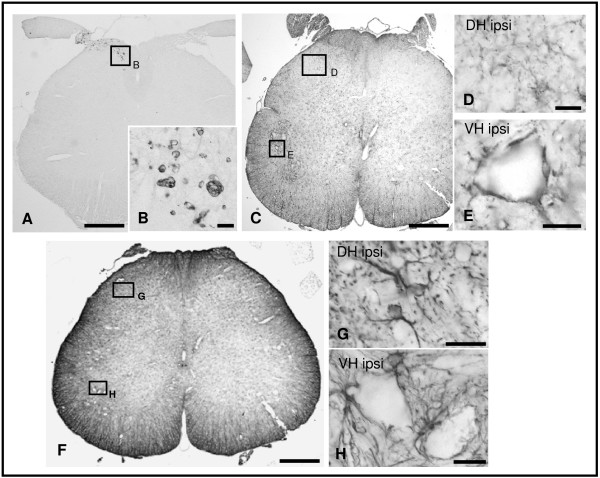
**Reactive gliosis in the lumbar SC 14 d after L5 ligation and transection**. ED1-immunopositive macrophages distributed within the fasciculus gracilis (A, B), while a higher density of PT66-immunopositive microglial cells (C-E) and GFAP-immunopositive astrocytes (F-H) is also found in the ipsilateral dorsal (DH; D, G) and ventral horn (VH; E, H). Scale bars, 500 μm (A, C, F), 20 μm (B, D, E, G, H).

Concurrent with the upregulation of CATS and CATX a strong gliosis appeared in the affected regions. In the ipsilateral fasciculus gracilis we observed numerous ED1-immunopositive macrophages (Fig. 5A, B), while in the ipsilateral DH and VH PT66-immunopositive microglia and GFAP-immunopositive astrocytes were more abundant than in the contralateral side (Fig. 5C–H).

**Figure 6:**
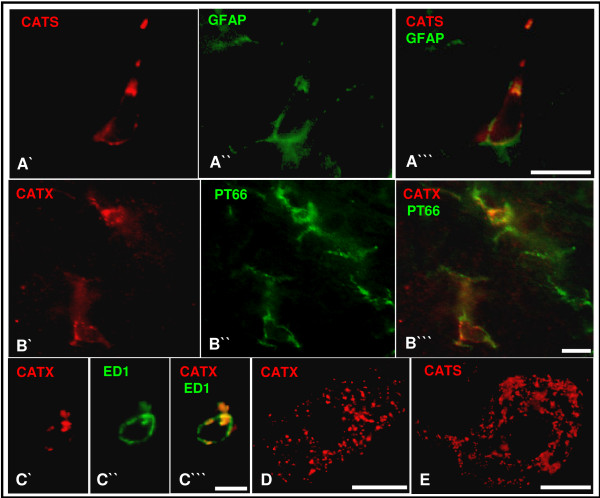
**Phenotyping of CATS and CATX cells**. Double-immunfluorescence of the spinal cord shows colocalization of CATS with the astrocyte marker GFAP (A'-A"') and colocalization of CATX with the microglial marker PT66 (B'-B"') and the macrophage marker ED1 (C'-C"'). Large motoneurons expressed CATX (D) and CATS (E). Scale bars, 10 μm (A, B), 5 μm (C), 20 μm (D, E).

To determine the phenotype of CATS and CATX cells in vivo we performed double immunofluorescence. The majority of CATS-immunopositive cells expressed the microglia-marker PT66 or the astrocyte-marker GFAP (Fig. 6A'–A"'), while only a small number of neurons were CATS-immunopositive (Fig. 6E). In contrast, CATX-immunoreactivity colocalizes only with single glial cells (Fig. 6B'–B"') but was more abundant in neurons (Fig. 6D). All ED1-immunopositive macrophages expressed both proteins (Fig. 6C'–C"').

## Discussion

The dorsal spinal cord is the first relay station in sensory perception, which receives, transmits and modulates the signals from peripheral nerves. Recent research has uncovered that peripheral nerve injury triggers cascades of systemic, cellular and molecular changes [4,33]. Moreover, there is ample evidence that these dynamic changes contribute to prolonged abnormal pain sensations. In the current study we analysed the participation of two cysteine proteases, the CATS and CATX, in the molecular processes underlying the induction and maintenance of neuropathic pain. Our results clearly show that immediately after surgery, concurrent to the onset of mechanical allodynia, both proteases, CATS and CATX, are upregulated in the spinal cord. Moreover, the upregulation of CATX protein was accompanied by a substantial increase in activity. In contrast to the increase in the proform level that of the CATX activity was highly significant. This apparent contradiction might be explained by either an increase of the active form of CATX, that is still below the detection level of the Western Blot, or a concomitant regulation of endogenous cathepsin inhibitors, the cystatins or thyropins [34,35]. In fact there is recent evidence in favor of the idea that the cathepsin inhibitors are also regulated during persistent pain states [36].

Both, CATS and CATX, are widely expressed in the brain [26] and have been implicated in several neurological conditions such as Alzheimer's disease [26,37,38], amyotrophic lateral sclerosis [26,27] and age-related inflammation [26]. Recently, CATS has also been implicated in neuropathic hyperalgesia and allodynia. Using the gene chip technology CATS mRNA was found to be upregulated in the ipsilateral L4 and L5 DRG in the PSL and CCI model [11], while in the SNL or L5T model, CATS mRNA expression was regulated in the DRG [39,40] and in the spinal cord [9,10]. Extending the latter results, we were able to detect the regulation of CATS protein levels and added a new cathepsin, CATX, to the list of regulated proteases in pain pathology.

Our immunohistochemical analysis, following the temporal development of neuropathic pain, supports the view that the upregulation of CATS and CATX expression is dynamic and proceeds along the fasciculus gracilis up to the medulla oblongata. Immediately (1 d) after transection CATS and CATX expressions increase. For both proteins this increase is restricted to the fasciculus gracilis, the dorsal horn and the layer IX in the ventral horn in the lumbar segment. As early as 2 d after injury the upregulation in the fasciculus gracilis spread cranially and reached the gracile nucleus at 1 w after injury. At that time point this nucleus exhibited morphological signs of degeneration. We never found cathepsin upregulation on the contralateral side of the lesion or in sham operated animals. This characteristic spatio-temporal pattern suggests that the upregulation of CATS/X expression accompanies the degenerative process of the transected axons [41].

Whether the cathepsins contribute to the mechanism of degeneration and are causally involved in the pain processing or whether the differential expression is an epiphenomenon is difficult to answer on the basis of the data provided here. However, there is recent evidence that CATS is directly involved in the pain process by modulating the cytokine response [28]. Cathepsins display rather diverse physiological actions. CATS for instance is well recognized for its crucial function in the control of antigen presentation [31] and its role in the degradation of the extracellular matrix. In contrast to CATS, little is known about the physiological function of CATX. But the high expression of CATX in antigen-presenting monocytes/macrophages [42,43], glial cells [26] and dendritic cells [32], its upregulation in the gastric mucosa of patients with *Helicobacter pylori *gastritis [44] and in the plasma of patients with multiple trauma [45] as well as its involvement in the production of bradykinin potentiating peptide [46] also imply a role in inflammatory processes.

## Conclusion

Our results suggest a strong regulation of both, CATS and CATX, in the spinal cord of an animal model of neuropathic pain. Whether the cathepsins contribute to the mechanism of degeneration and are causally involved in the pain processing or whether the differential expression is an epiphenomenon is difficult to answer on the basis of the data provided here. Further experiments such as application of specific cathepsin inhibitors are required before the exact role of single cathepsin subtypes in the pain process can be unraveled.

## Methods

### Surgery

Male Wistar rats (Janvier, Le Genest Saint Isle, France) with a weight of 200–250 g were used. Animals were housed in a climate-controlled room on a 12–12 light-dark cycle. Food and water were available *ad libitum*. All procedures were approved by the local animal usage committees according to German guidelines on animal care and use.

Prior to the operation, rats were deeply anesthetized with pentobarbitone at a dose of 50 mg/kg i.p. The L5T model was achieved by transection of the left L5 spinal nerve in a procedure modified from Kim and Chung [47,48]. In sham controls the sciatic nerve was exposed but not transected.

Rats were sacrificed at day 1 (1 d) – 35 d for Western Blot analysis (L5T n = 5, sham n = 5), immunohistochemistry (L5T n = 2–4, sham n = 4) and activity assays (L5T n = 4, sham n = 7, naïve n = 4).

### Behavioral tests

Withdrawal tests for evaluation of tactile allodynia were measured by the use of the dynamic plantar aesthesiometer. The animals were placed into raised plexiglass boxes with mesh flooring and allowed to acclimatize for at least 15 min until exploratory behavior ceased. Sampling was conducted by a metal filament which was applied manually to the ventral mid-plantar hind paw. The force raised (0–50 g) with time (20 s) until the rat lifted its paw. The mean withdrawal threshold for both hind paws was taken from a set of three applications, not less than 2 min apart.

### Tissue preparation

For immunoblotting, spinal cord and brain tissues were homogenized in triple detergent lysis buffer (50 mM HEPES pH 7.4, 150 mM NaCl, 10 mM EDTA, 1% Nonidet P40, 0.5% sodium deoxycholate, 0.1% sodium dodecyl sulfate, complete protease inhibitor cocktail (Roche Applied Science, Mannheim, Germany) using a Teflon/glass homogenizer at 4°C. Homogenized samples were kept on ice for 30 min und subsequently centrifuged for 10 min in a precooled centrifuge at 12,000 g. The supernatant was collected and subsequently diluted 1:1 in 2× Laemmli sample buffer and boiled for 5 min. Protein determination was performed by the method of Neuhoff and coworkers [49].

For immunohistochemistry, animals were transcardially perfused with phosphate-buffered saline (PBS) followed by 4% paraformaldehyde (PFA) in 0.1 M phosphate buffer (PB). The brain, the spinal cord and the ipsi- and contralateral nerves L4–L6 were excised and postfixed for 24 h in the perfusion fixative. Spinal cords were subdivided into the spinal cord segments according to the numbers of the related spinal nerves and all segments and the L5 (ipsi- and contralateral) nerves were embedded in paraffin. Serial, transversal 18-μm-thick sections were cut throughout all spinal cord segments, the hindbrain and the peripheral nerves. Sections were mounted on Superfrost slides (Carl Roth, Karlsruhe, Germany).

For CATX activity assays, the tissues were thawed separately on ice and homogenized in 7-fold volume (w/v) of 100 mM NaCl, 50 mM NaOAc, 4 mM EDTA-Na_2_, 0.1% Triton X-100, pH 5.0 [50]. All procedures were carried out at 4°C. After incubating the samples for 60 min on ice, they were centrifuged (60 min at 13,000 g) for elimination of debris. Supernatants were stored in aliquots at -80°C until further use. Protein contents of the preparations were measured by the method of Neuhoff [49].

### Western blots

Proteins were electrophoretically separated on a 10% polyacrylamide gel containing SDS and transferred onto a PVDF-membrane (Carl Roth, Karlsruhe, Germany) at 4°C with 200 mA for 1.5 h. Blocking was performed with 1.5% milk powder and 1% BSA in TBS-Tween (0.1% Tween, 20 mM TBS) at RT for 1 h. Incubation with the primary antibodies goat anti-mouse CATX (1:500; R&D Systems, Wiesbaden, Germany) or goat anti-human CATS (1:200; R&D Systems) was conducted in blocking buffer overnight at 4°C. The next day blots were incubated in HRP-coupled anti-goat (1:50,000 in 1.5% milk powder in TBS-Tween; Amersham Biosci., München, Germany) at RT for 1.5 h, followed by detection with the ECL-Plus system (Amersham Biosci.).

To control protein loading, the blots were incubated with mouse anti-chick α-tubulin (1:400,000; Sigma, Deisenhofen, Germany) followed by HRP-coupled anti-mouse (1:50,000 in 1.5% milk powder in TBS-Tween; Amersham Biosci.) and the ECL-Plus system as described above.

### Immunohistochemistry

#### Antibodies

Primary antibodies were used to document CATS and CATX and to identify the cathepsin-expressing cells. We used the following antibodies: goat anti-rat CATS (anti-CATS; 1:100–200, Santa Cruz Biotechnology, Santa Cruz, USA suitable for detection of prepro-, pro- and mature form of CATS); goat anti-mouse CATX (anti-CATX; 1:100–200; R&D systems, Abingdon, UK; suitable for detection of prepro-, pro- and mature form of CATX), the microglia/macrophage marker mouse anti-phosphotyrosine (anti-PT66; 1:1000; Sigma), the astrocyte marker mouse anti-glial fibrillary acidic protein (anti-GFAP; 1:2500–5000; Chemicon, Hampshire UK) and the macrophage marker mouse anti-rat CD68 (anti-ED1; 1:5000; Serotec, Düsseldorf, Germany).

#### Stainings

Single immunohistochemical stainings were performed on deparaffinized sections after antigen retrieval (5 min cooking in 0.01 M citrate buffer pH 6.0, for all primary antibodies except ED1). Following the first antiserum incubations, sections were treated by the corresponding biotinylated secondary antibody (Axxora, Lörrach, Germany), and the ABC reagent (Axxora). Peroxidase reaction was carried out with 3,3'-diaminobenzidine as the chromogene and intensified with silver-gold [51]. Specificity of the stainings was either confirmed by omitting primary antibodies or by preabsorption with a five-fold (by weight) excess of specific blocking peptides for 2 h at RT (for anti-CATS and anti-CATX) (Fig. 1G and 1I).

Immunofluorescence double IHC was performed on deparaffinized sections after antigen retrieval. Therefore, we labeled sections simultaneously with primary antibodies and subsequently incubated them with biotinylated secondary antibody (1:300; Axxora) followed by fluorescein isothiocyanate-labeled Avidin (1:400; Axxora) and CY3-labeled secondary antibody (1:500; Dianova, Hamburg, Germany).

### CATX activity assay

Spinal cord tissues were used for CATX enzyme activity tests. CATX activity was measured in 25 mM CH_3_COONa/1 mM EDTA/5 mM DTT (pH 3.5) with 10 μM MCA-R-P-P-G-F-S-A-F-K(Dnp)-OH (R&D Systems) as substrate. In parallel assays, the specific CATB inhibitor CA-074 (1 μM, Bachem, Weil am Rhein, Germany; Ki = 2 nM for purified rat CATB and 40–200 μM for CATH and CATL; [52]) and the non-specific cysteine protease inhibitor E-64 (5 μM, Sigma) were added in order to distinguish between CATB activity and the entire cysteine protease activity. A clear determination of CATB activity is essential as CATB is also able to hydrolyze the substrate at these conditions. Activity of other proteases being able to cleave MCA-R-P-P-G-F-S-A-F-K(Dnp)-OH, like CATL and ECE-1, was undetectable (data not shown). Assays were performed at RT in black 96 multiwell plates (Falcon) in a total volume of 50 μl. Prior starting the assay by addition of substrate, the enzyme solutions were pre-incubated for 5 min with the different inhibitors or diluent at RT. After a 30 min incubation time the assays were stopped by the addition of 50 μl stop solution (100 mM CH_2_ClCOOH, 70 mM CH_3_COOH, 30 mM CH_3_COONa, pH 4.3) and measured at 340/430 nm, according to the procedures described by [53]. The activities of the proteases were calculated on the basis of relative fluorescent units. The total cathepsin activity was assessed by taking the difference between non-inhibited and E-64 inhibited samples. CATB activity corresponded to the part of the response that could be inhibited by CA-074. The difference between the fluorescence measured in the presence of the CATB inhibitor and the fluorescence measured in the presence of E-64 was attributed to CATX activity.

### Data analysis

#### Animals

**Figure 7:**
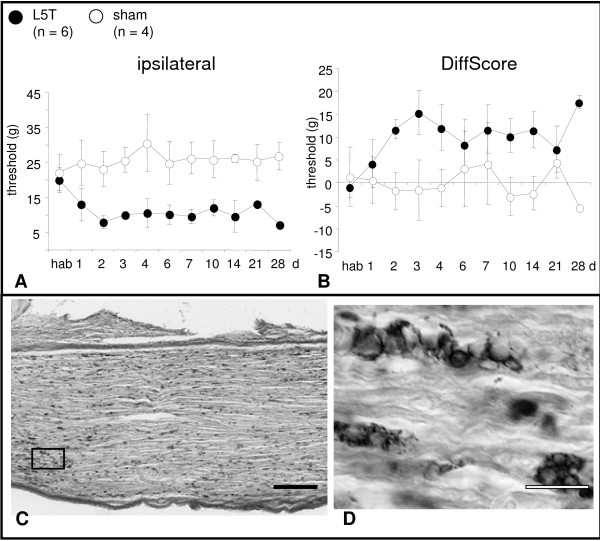
**Behavior and histopathology after L5 transection**. **A, B: **Time course of neuropathic mechanical allodynia in the ipsilateral hind paw expressed as ipsilateral threshold (A) and Diffscore (B, difference between contralateral and ipsilateral withdrawal threshold) in L5T and sham operated animals. The L5T lesion led to a pronounced mechanical allodynia for up to 28 d. **C, D: **Representative example of ED1-immunostained proximal stump of transected L5 at 14 d after injury. The whole stump is densely filled with ED1-immunopositive macrophages (C) aligning along the trajectory of the axons (D). hab, habituation. Scale bars, 200 μm (C) and 20 μm (D).

Animals included in the present study fulfilled the following behavioral and anatomical criteria: (i) strong mechanical allodynia measured by the aesthesiometer and defined as an ipsilateral threshold difference between pre- and post-surgery of at least 5 g (Fig. 7A) and a DiffScore (contralateral threshold minus ipsilateral threshold) of a minimum of 5 g (Fig. 7B) and (ii) persistent L5 transsection (short survival times) and/or infiltration of L5 by numerous ED1-immunopositive macrophages (long survival times) (Fig. 7C, D).

#### Western blots

Band intensities were quantified using the analysis software TINA 2.09 (raytest Isotopenmeßgeräte GmbH, Straubenhardt, Germany) and normalized relative to the intensity of the corresponding α-tubulin bands. All data are presented as relative percentage of the means ± SD. Statistical significance was determined using the Student's t-test. P-values of less than 0.05 were considered to be statistically significant.

#### Immunohistochemical stainings

Peroxidase-labeled immunohistochemistry sections were visualized at the microscopic level (Axioskop2; Zeiss, Oberkochen, Germany) under brightfield illumination and Nomarski optics, while fluorescent structures were analyzed by epifluorescence (Axioskop2). Structures were identified with the aid of the atlas of [54]. The anatomic terminology used in this study is based on this atlas. Images were captured with an imaging system (JVC, KY-F75U camera) connected to a computer equipped with an image program (Diskus 4.50, Hilgers, Königswinter, Germany).

Alterations of staining intensity or distribution of stained structures of each animal were independently analysed by two examiners blind with respect to the treatment of the animals.

#### Cathepsin activity

Data obtained from the CATX activity assays were analyzed by means of one-way ANOVA to determine statistical significance. All pairwise multiple comparison procedures were performed by Tukey's *post hoc *test.

## Abbreviations

C: cervical; CATS: cathepsin S; CATX: cathepsin X; DH: dorsal horn; DRG: dorsal root ganglia; FG: fasciculus gracilis; L: lumbar; L5: 5^th ^lumbar nerve; L5T: ligation and transection of L5; NG: nucleus gracilis; SC: spinal cord; T: thoracic; VH: ventral horn.

## Authors' contributions

AL and IB carried out the L5T surgeries and behavioral analysis, the immunohistochemical analysis and the activity assays; WW established the activity assays; MA helped with L5T surgeries; BS identified the cathepsin overexpression and performed preliminary experiments; CCS and HL conceived the study and oversaw all components including manuscript preparation. All authors read and approved the final manuscript.

## Supplementary Material

Additional file 1 Upregulation of cathepsin protein levels after L5T. Western blot analyses of CATX and CATS proform expression in the spinal cords of sham (n = 5) and L5T (n = 5) operated rats at 8 d after injury. Cervical, thoracic and lumbar segments were analyzed. Each band represents a single animal. C, cervical; T, thoracic; L, lumbar. Click here for file

Additional file 2 Quantification of Western blot analyses. Quantification of Western blot analyses of CATX and CATS proform expression in the spinal cord of L5T (n = 4) and sham operated animals (n = 4) (repetition of the experiment #1 – shown in the paper). Expression levels were normalized relative to the corresponding α-tubulin band. Similar to experiment #1 – the histogramms show an upregulation of both cathepsins in all SC segments. C, cervical; T, thoracic; L, lumbar. Click here for file
