# Supplementary material for: Differential expression of Cathepsin S and X in the spinal cord of a rat neuropathic pain model
Source: BMC Neurosci. 2008 Aug 12;9:80. doi: 10.1186/1471-2202-9-80 (PMC2527007; doi:10.1186/1471-2202-9-80)
Supplement: Additional file 1 — Upregulation of cathepsin protein levels after L5T. Western blot analyses of CATX and CATS proform expression in the spinal cords of sham (n = 5) and L5T (n = 5) operated rats at 8 d after injury. Cervical, thoracic and lumbar segments were analyzed. Each band represents a single animal. C, cervical; T, thoracic; L, lumbar. [file 1471-2202-9-80-S1.pdf]

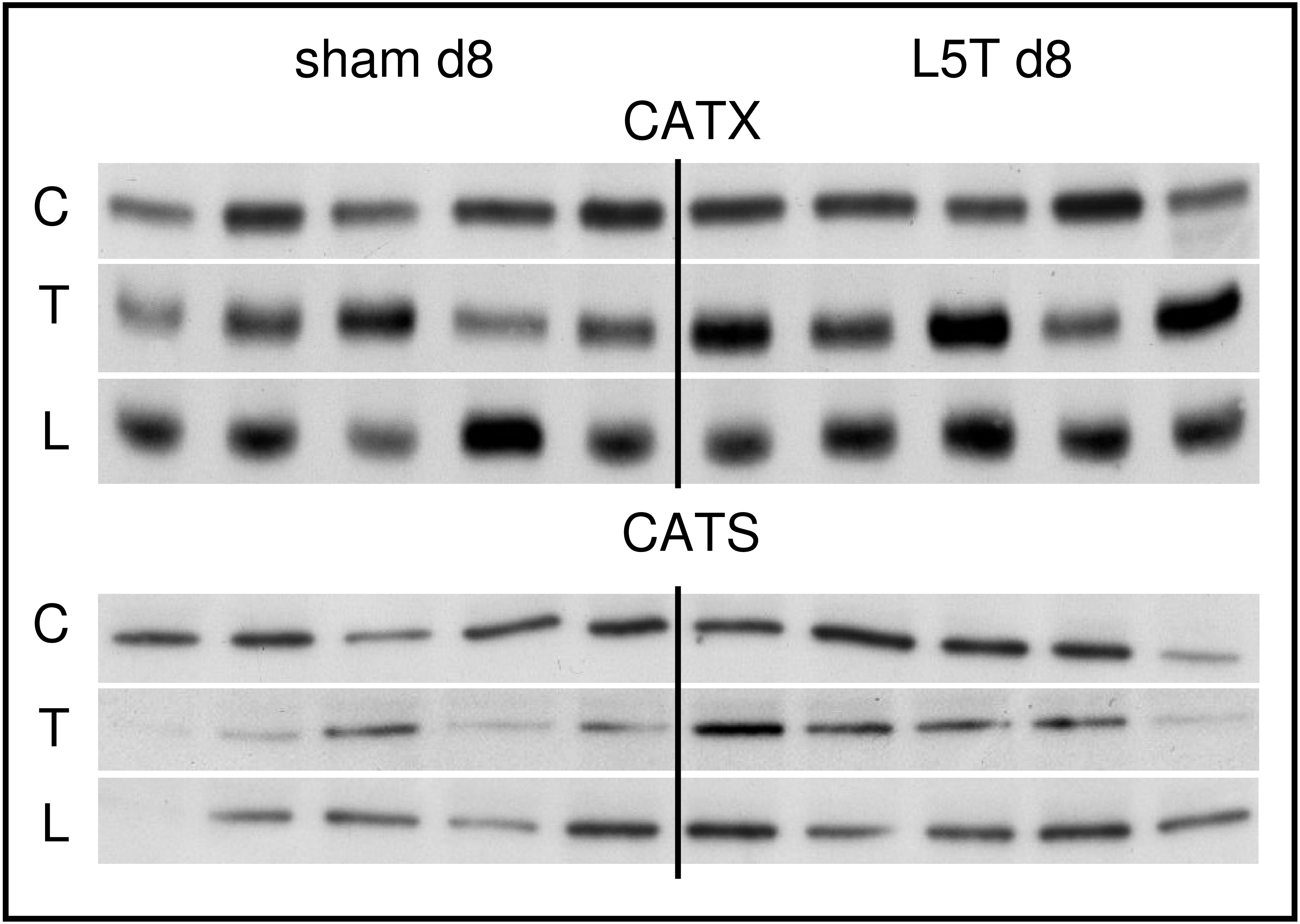
sham d8
L5T d8
CATX
C
T
L
CATS
C
T
L
