# Supplementary figures and images for: Differential expression of Cathepsin S and X in the spinal cord of a rat neuropathic pain model
Source: BMC Neurosci. 2008 Aug 12;9:80. doi: 10.1186/1471-2202-9-80 (PMC2527007; doi:10.1186/1471-2202-9-80)

CATX

CATS

□ L5T  
■ sham

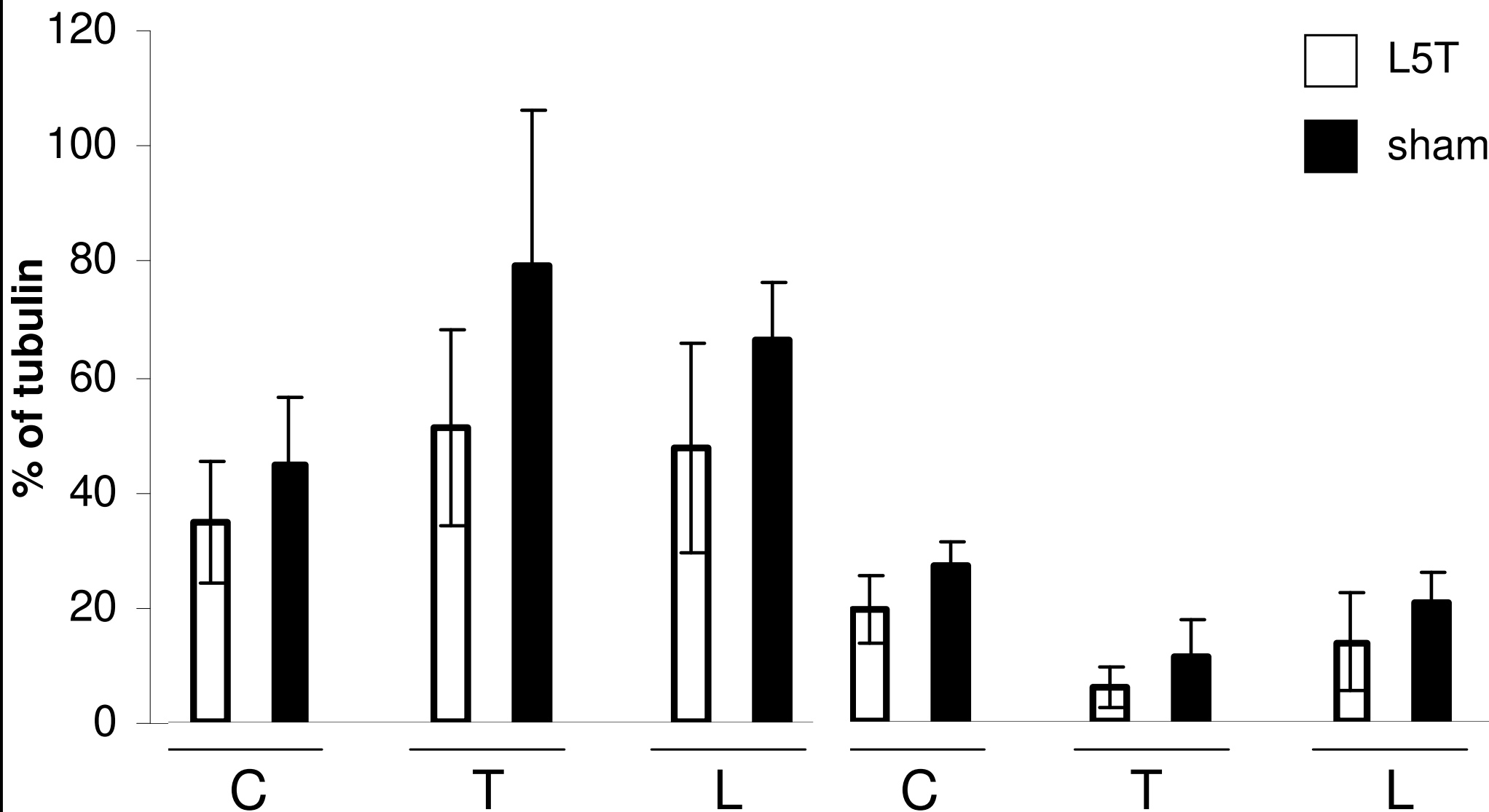

Supplement: Additional file 2 — Quantification of Western blot analyses. Quantification of Western blot analyses of CATX and CATS proform expression in the spinal cord of L5T (n = 4) and sham operated animals (n = 4) (repetition of the experiment #1 – shown in the paper). Expression levels were normalized relative to the corresponding α-tubulin band. Similar to experiment #1 – the histogramms show an upregulation of both cathepsins in all SC segments. C, cervical; T, thoracic; L, lumbar. [file 1471-2202-9-80-S2.pdf]
